# Influence of the shape of the first metatarsal cuneiform joint on the development of hallux valgus deformity

**DOI:** 10.1186/s12891-023-06668-4

**Published:** 2023-07-04

**Authors:** Mirko Sovilj, Andreja Baljozović, Filip Pilipović, Maja Sovilj Banjac, Zoran Baščarević

**Affiliations:** 1 Faculty of Medicine Belgrade, Belgrade, Serbia; 2 Institute of Orthopedics “Banjica” Belgrade, Andreja Baljozović, Belgrade, Serbia; 3 Clinical Hospital Center “Zvezdara” Belgrade, Belgrade, Serbia

**Keywords:** Hallux valgus, Etiology, The first metatarsal-cuneiform joint

## Abstract

**Objective:**

To examine the presence of certain shapes of the first metatarsal-cuneiform joint (MTC) joint in feet with hallux valgus (HV) deformity. To determine whether the anatomical orientation of this joint affects the size of the hallux valgus angle (HVA) and the first intermetatarsal angle (IMA) and whether it contributes to the dynamics of the developmental course of HV deformity.

**Methods:**

The shape of the first MTC joint was determined on a sample of 315 feet with HV deformity. The influence of the shape of this joint on the values of HVA and IMA was explored. The relation between the position of the tibial sesamoid and the size of HVA and IMA as well as the dynamics of the development of this deformity depending on the shape of the first MTC joint, was examined.

**Results:**

The oblique shape of the first MTC joint was found in 165 (52.4%) feet, the transverse in 145 (46%), and the convex shape was registered in five feet (1.6%). In the oblique shape of this joint, a moderate and severe degree of HV deformity is predominant, while in the transverse shape a mild degree dominates. A statistically significant dependence of HVA on the shape of the first MTC joint was found (Sig. = 0.010), while the dependence of IMA did not show statistical significance (Sig. = 0.105). HVA values follow the position of the tibial sesamoid in both shapes of the MTC joint while the size of the IMA in the transverse shape does not follow the change of the position of this sesamoid.

**Conclusion:**

The oblique shape of the first MTC joint is associated with the more severe form of HV deformity and its faster developmental course. In the analyzed sample, it was shown that HVA is higher in the oblique shape of the MTC joint and significantly depends on the anatomical orientation of this joint. Furthermore, IMA has a higher value in the oblique shape compared to transverse but this dependence is not statistically significant. The analysis showed that the oblique shape of the first MTC joint contributes to the development of HV deformity.

## Introduction

Most researchers believe that hallux valgus is a multifactorial caused deformity but that genetic predisposition is of particular importance [1, 2]. Since the first metatarsal (MT) bone has no grasp of tendon and ligament structures, except at its base, it is anatomically unstable [3, 4] which is why the shape of the first metatarsal-cuneiform joint (MTC) is essential and, as a consequence, its stability [5]. The intensity of medial displacement of the head of the first MT bone is indicated not only by increased angle between the axes of the first and second MT bones (IM angle), but also by position of medial sesamoid in relation to the axis of the first MT bone that gradually abandons the sesamoid apparatus.

In 1925, Truslow proposed the term metatarsus primus varus, which was also supported by Lapidus (1934), understanding that the movement of the first MT bone toward the midline of the body is a major feature of HV deformity. In phylogenetic development, the foot has evolved from a gripping function favored by a greater degree of stiffness, and an MTC joint with a greater range of motion, to a static function of transferring body weight to the ground, and dynamic when rejecting the body from the ground when walking, which requires a firm lever [4, 6–8]. The oblique shape of the first MTC joint with different degrees of medial obliqueness contributes to the increase of the first IM angle and thus to the further development of hallux valgus deformity [7, 9–11]. Doty et al. [12] concluded that an increase in the medial inclination of the MTC joint may be associated with an increase in the IM angle while Dayton et al. [13] confirm a linear relationship between the MTC angle and the IM angle but without a sufficient degree of significance. Anatomical research identified three types of MTC joints depending on the number of separate joint veneers, with the fact that three facets were found only in feet without HV deformity [14]. For radiographic definition of the first MTC joint, we have several different angle measurements formed by the line of the distal articular surface of the first cuneiform bone with: axis line I or II MT bone, the medial or lateral edge of the body of the first cuneiform bone [4, 9–12, 15, 16], and Chopart joint line [13]. Hence, there is no harmonized position regarding the measurement of radiographic parameters of the MTC joint and therefore in our research, we opted for a pragmatic approach by determining the shape of the first MTC joint based on the radiographic image.

## Materials and methods

An observational study was conducted in the form of a descriptive-analytical study in which 269 patients and 396 surgically treated feet with severe hallux valgus deformity were treated at the Institute of Orthopedics ‘Banjica’ in Belgrade in the period from 1993 to 2010. At the admission, all patients agreed that the medical documentation on their treatment could be used for research purposes. For persons under the age of 18, informed consent was given by their parents or guardians. All applied procedures of this study were approved by the Institute. The consent of the Ethics Committee of the Institute of Orthopedics “Banjica” Belgrade for this study was also obtained. All methods used in the research were carried out in accordance with relevant guidelines and regulations.

**Fig. 1:**
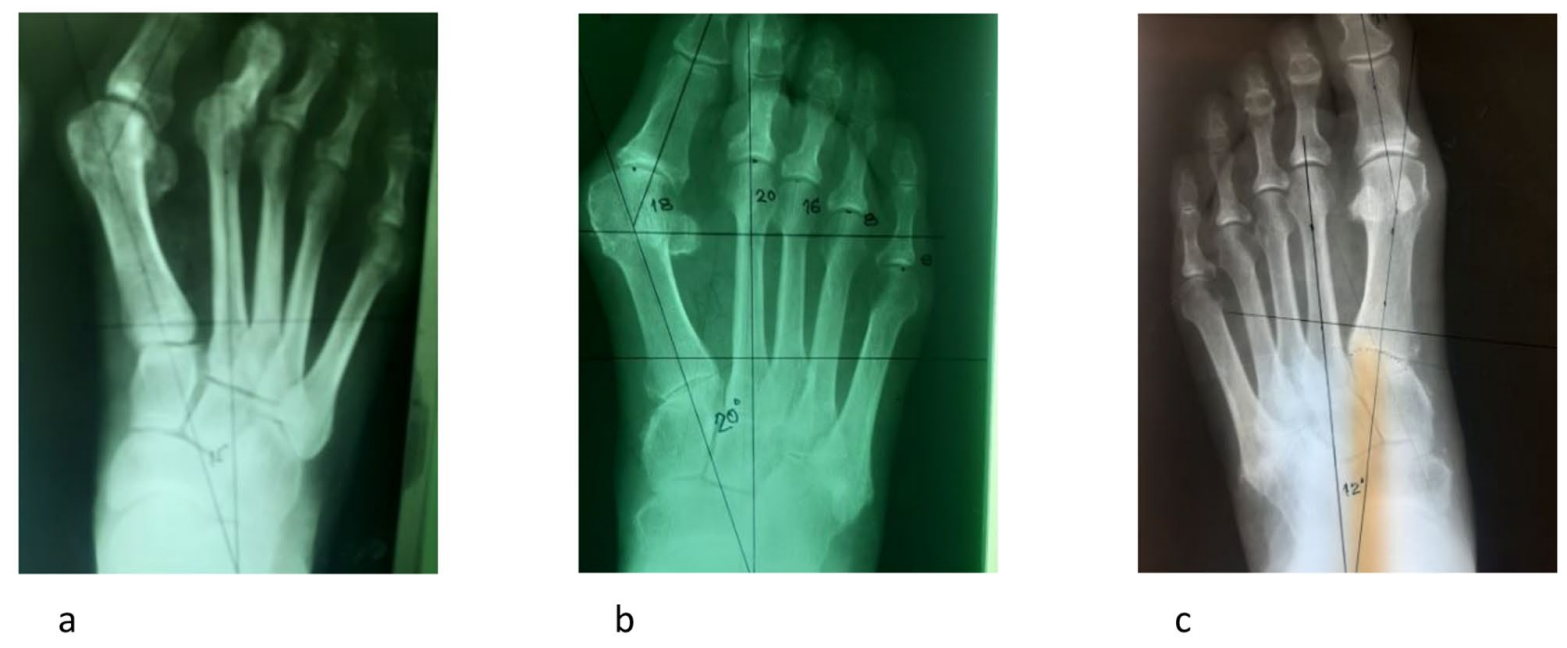
Three shapes of the first metatarsal-cuneiform joint : **(a)** transverse, **(b)** oblique, **(c)** convex

On radiographs of the foot under load, with an inclination of the X-ray tube of 15 degrees in relation to the vertical and at a distance of 1 m, measurements of HVA, IMA was performed, the position of the tibial sesamoid in relation to the axis and I MT of the bone was defined, and the shape of the I MTC joint was determined. Thus, it was determined whether it is a transversely placed position of joint surfaces that are parallel to the line perpendicular to the axis of the II MT bone with a tolerance of up to 5 degrees (Fig. 1.a) or with the previously mentioned line form a larger angle when we defined it as the oblique shape of the I MTC joint (Fig. 1.b). We also registered the third form with an emphasized convex shape of the distal articular surface of the first cuneiform bone (Fig. 1.c). Excluded from this study were cases that had previously undergone osteoarticular surgical treatment or had previously had injuries to the bone and joint structures of the feet, suffering from rheumatism, diabetes, or neuromuscular disease. Based on the stated criteria, 81 cases were excluded, so that further study was conducted on 315 feet.

For the purposes of analyzing the influence of the MTC joint shapes on the development of hallux valgus, the deformities were grouped into three groups: - mild deformity (HVA < 30 and IMA < 13 degrees); -moderate deformity (HVA < 40, IMA < 20 degrees); -severe deformity (HVA > 40, IMA > 20 degrees) [17] .

In order to specify the relationship of the head and MT bone to the sesamoid mechanism, four positions of the tibial sesamoid are defined: ‘0’ anatomical position, ‘I’ tibial sesamoid crosses the axis up to 50% of the volume, ‘II’ tibial sesamoid crosses the axis over 50%, ‘III’ The tibial sesamoid crosses the axis of the I MT of the bone in its entire circumference [17] .

In order to more accurately monitor the changes in the deformity, the subjects were grouped into five groups according to their age: persons younger than 18, 19 to 32, 33 to 46, 47 to 59, and 60 and older. For the same reason, five groups of subjects were formed depending on the length of the period of development of the deformity, assuming that this is the period from the onset of symptoms to the indicated indications for surgical treatment: group 1 (up to five years), group 2 (six to 10), group 3 from 11 to 15), group 4 (from 16 to 20) and group 5 with 21 years and older.

## Results

Of the 315 treated feet, 312 (99.1%) belonged to women and only 3 or 0.9% to men. The average age of the patients included in the study was 45.09 years with SD = 13.23, while the average age at the onset of symptoms related to this deformity was 36.19 years with SD = 12.15. The period of deformity development is on average 8.9 years with SD = 4.61. The mean value of the measured HVA is 35.57 degrees with SD = 7.33 with a value range of 16 to 61 degrees, while the mean IMA value is 13.88 with SD = 2.88 and a value range of 10 to 27 degrees. The transverse shape of the first MTC joint was found in 145 or 46% of the feet, convex in 5 or (1.6%) while the oblique shape of this joint was most common, in 165 or 52.4% of cases. The ‘III’ position of the tibial sesamoid was most often determined in 260 or 82.5% of the feet, the ‘II’ position in 50 or 15.9% of the feet, while the ‘I’ position was determined in three cases or 1%.

**Table 1:** Structure of HV deformity measured by HVA values in established shapes of the I MTC joint

		Deformity measured by HVS value			${\varvec{\chi }}^{2}$(df. N) Sig.
		**Mild deformity N = 114 (36,2)**	**Moderate deformity N = 127 (40.3%)**	**Severe deformity** **N = 74,(23.5%)**	
Shape of the metatarsal-cuneiform joint	Transversal N = 145,(46%) Me = 32.59,SD = 7.106, 95%CI:od 31.43–33.76, Min = 16,Max = 50	64(56.1%) 44.1%	50(39.4%) 34.5%	31(41.9%) 21.4%	${\chi }^{2}$(4.315) = 13.332, Sig.=0.010
	Convex N = 5, (1.6%) Me = 26.60,SD = 3.847 95%CI:od 21.82–31.38 Min = 22,Max = 31	4(3.5%) 80.0%	1(0.8%) 20.0%	0 (0%) 0.0%	
	Oblique, N = 165,(52.4%) Me = 34.65,SD = 7.40 95%CI:od 33.51–35.79 Min = 18,Max = 61	46 (40.4%) 27.9%	76(59.8%) 46.1%	43(58.1%) 26.1%	
ANOVA		F(2,312) = 5.482, Sig.=0.005			

95%CI – 95% confidence interval for the estimated mean value

In the transverse shape of the first MTC joint, the highest percentage is mild deformity (44.1%) and then moderate in 34.5%, while in the oblique shape the most common is moderate deformity (46.1%) and then mild in 27, 9%. Severe deformity is present in the oblique shape in 26.1%, and in the transverse form in 21.4%. In the convex shape, four cases are mild and one is moderate. Pearson’s Chi square independence test showed that there was a statistically significant relationship between HVA and shapes of the MTC joint; χ ^ 2 (4,315) = 13,332, Sig. = 0.010 (Table 1).

**Table 2:** Structure of HV deformity measured by IMA values in established shapes and MTC of the joint

		Deformity measured by IMU value			${\varvec{\chi }}^{2}$(df. N) Sig.
		Mild deformity N = 158,(50.2%)	Moderate deformity, N = 145(46.0%)	Severe deformity N = 12(3.8%)	
Shape of the metatarsal-cuneiform joint	Transversal N = 145, (46%) Me = 13,55, SD = 2.66, 95%CI:od 13.11–13.99, Min = 10,Max = 23	81(51.3%) 55.9%	59(40.7%) 40.7%	5(41.7%) 3.4%	${\chi }^{2}$(4.315) = 7.666, Sig.=0.105
	Convex N = 5, (1.6%) Me = 15.80,SD = 3.962, 95%CI:od 10.88–20.72 Min = 10,Max = 21	1(0.6%) 20.0%	3(2.1%) 60.0%	1(8.3%) 20.0%	
	Oblique, N = 165,(52.4%) Me = 14.12,SD = 2.997, 95%CI:od 13.65–14.58 Min = 10,Max = 27	76(48.1%) 46.1%	83(57.2%) 50.3%	6(50.0%) 3.6%	
ANOVA		F(2,312) = 2.636, Sig.=0.073			

Analysis of the distribution of HV deformity measured by IM angle displayed a more even representation according to its severity compared to the shape of the I MTC joint. Pearson’s Chi square independence test shows that the dependence of IMU on the shape of the MTC joint is not statistically significant, χ ^ 2 (4,315) = 7,666; Sig. = 0.105. (Table 2)

**Table 3:** Distribution of subjects according to the severity of the deformity measured by HVA values in relation to the shape of the first MTC joint and the position of the tibial sesamoid

MTC joint shape			Deformity measured by HVA value			Sig.
			**Mild deformity**	**Moderate deformity**	**Severe deformity**	
Transversal	Position of the tibial sesamoid	First,N = 2 (1.4%) Me = 23.0, SD = 7.071, Min = 18,Max = 28	2(3.1%) 100.0%	0(0%) 0.0%	0(0%) 0.0%	0.002
		s,N = 23 (15.9%) Me = 26.17, SD = 6.239, Min = 16,Max = 40	18(28.1%) 78.3%	4(8.0%) 17.4%	1(3.2%) 4.3%	
		Third,N = 120 (82.8%) Me = 33.98, SD = 6.466, Min = 19,Max = 50	44(68.8%) 36.7%	46(92.0%) 38.3%	30(96.8%) 25.0%	
	Total, N = 145 (100%)Me = 32.59, SD = 7.106, Min = 16,Max = 50		64 (44.1%)	50 (34.5%)	31 (21.4%)	
ANOVA			**F(1,142) = 16.391,Sig.=0.000, Eta squared = 0.188**			
Convex	Position of the tibial sesamoid	Second,N = 1 (20.0%), Me = 22.00, SD = 0.00	1(25%) 100.0%	0(0%) 0.0%		0.576
		Third,N = 4 (80%),Me = 27.75, SD = 3.304,Min = 24,Max = 31	3(75%) 75.0%	1(25%) 25.0%		
	Total,N = 5,(100%), Me = 26.60,SD = 3.847, Min = 22,Max = 31		4 (80.0%)	1 (20.0%)		
ANOVA			F(1,3) = 2.423,Sig.=0.217			
Oblique	Position of the tibial sesamoid	First,N = 1,(0.6%) Me = 24,00, SD = 0.00	1(2.2%) 100.0%	0 (0%) 0.0%	0(0%) 0.0%	0.000
		s,N = 26 (15.8%),Me = 28.15 SD = 6.182,Min = 18,Max = 40	17(37.0%) 65.4%	8(10.5%) 30.8%	1(2.3%) 3.8%	
		Third N = 138 (83.6%),Me = 35.95, SD = 6.931,Min = 19,Max = 61	28(60.9%) 20.3%	68(89.5%) 49.3%	42(97.7%) 30.4%	
	Total, N = 165 (100%), Me = 34.65 SD = 7.4, Min = 18, Max = 61		46 27.9%	76 46.1%	43 26.1%	
ANOVA			**F(3,161) = 11.123,Sig.=0.000, Eta squared = 0.172**			

There is no significant difference in the percentage of ‘II’ and ‘III’ position of the tibial sesamoid in the transverse (15.9% and 82.8%) and oblique (15.8% and 83.6%) shapes of the MTC joint. Pearson’s Chi square test showed that there is a statistically significant relationship between the position of the tibial sesamoid and HVA in both the transverse (Sig. = 0.002) and oblique shape of the MTC joint (Sig. = 0.000), (Table No. 3).

**Table 4:** Distribution of subjects according to the severity of the deformity measured by IMA values in relation to the shape of the first MTC joint and the position of the tibial sesamoid

MTC joint shape			Deformation measured by the value of the IM angle			Sig.
			**Mild deformity**	**Moderate deformity**	**Severe deformity**	
Transversal	Position of the tibial sesamoid	First,N = 2 (1.4%) Me = 12.0 SD = 1.414, Min = 11,Max = 13	2(2.5%) 100.0%	0(0%) 0.0%	0(0%) 0.0%	0.102
		s,N = 23 (15.9%) Me = 12.30, SD = 1.974, Min = 10,Max = 17	18(22.2%) 78.3%	5(8.5%) 21.7%	0(0%) 0%	
		Third,N = 120 (82.8%) Me = 13.82, SD = 1.987 Min = 10,Max = 23	61 (75.3%) 50.8%	54 (91.5%) 45.0%	5 (100%) 4.2%	
	Total,N = 145 (100%) Me = 13.55 SD = 2.661,Min = 10,Max = 23		81 (55.9%)	59 (40.7%)	5 (4.4%)	
ANOVA			**F(2,142) = 3.585,Sig.=0.030, Eta squared = 0.048**			
Konveksni	Position of the tibial sesamoid	Second,N = 1 (20.0%) ,Me = 15.00,SD = 0.00	0 (0%) 100.0%	1(33.3%) 0.0%	0	0.659
		Third,N = 4 (80%), Me = 16.45, SD = 4.546, Min = 10, Max = 21	1 (100%) 25.0%	2(66.7%) 50.0%	1 (100%) 25.0%	
	Total,N = 5,(100%), Me = 15.8,SD = 3.962,Min = 10,Max = 21		1 (20.0%)	3 (60.0%)	1(20%)	
ANOVA			F(1,3) = 0.039,Sig.=0.857			
Oblique	Position of the tibial sesamoid	First,N = 1,(0.6%) Me = 11,00,SD = 0.00	1(1.3%) 100.0%	0 (0%) 0.0%	0(0%) 0.0%	0.002
		s,N = 26 (15.8%),Me = 12.04 ,SD = 1.907,Min = 10,Max = 16	21(27.6%) 80.8%	5(6.0%) 19.2%	0(0%) 0%	
		Third,N = 138 (83.6%),Me = 14.53,SD = 3.004, Min = 10,Max = 27	54(71.1%) 39.1%	78 (94.0%) 56.5%	6(100%) 4.4%	
	Total, N = 165 (100%), Me = 14.12, SD = 2.997, Min = 10,Max = 27		76 (46.1%)	83 (50.3%)	6 (3.6%)	
ANOVA			**F(3,161) = 6.444,Sig.=0.000, Eta squared = 0.107**			

Pearson’s Chi square independence test showed that the dependence of the position of the tibial sesamoid and the IM angle in the oblique shape of the MTC joint was statistically significant (Sig. = 0.002), while in the transverse shape the stated dependence was not statistically significant (Sig. = 0.102). ) ( Table No. 4).

**Table 5:** Distribution of subjects according to the severity of deformity measured by HVA values in relation to the shape of the MTC joint and the period of deformity development

MTC joint shape			Deformity measured by HVA value			Sig.
			**Mild deformity**	**Moderate deformity**	**Severe deformity**	
Transversal	Period of deformity development	<= 5,(Group1) N = 30.(20.7%) Me = 28.63, SD = 7.285	20 (31.3%) 66.7%	8(16.0%) 26.7%	2(6.5%( 6.7%	0.013
		6–10 ,( Group 2) N = 73(50.3%) Me = 33.15, SD = 6.808	30(46.9%) 41.1%	26(52.0%) 35.6%	17(54.8%) 23.3%	
		11–15,( Group 3) N = 17(11.7%) Me = 33.06,Sd = 5.379	7(10.9%) 41.2%	8(16.0%) 47.1%	2(6.5%) 11.8%	
		16–20 (Group 4)N = 23 (15.9%) Me = 36.09,SD = 7.038	5(7.8%) 21.7%	8(16.0%) 34.8%	10(32.3%) 43.5%	
		21 and more (Group a 5): N = 2 (1.4%) Me = 27.50,SD = 3.536	2(3.1%) 100.0%	0(0%) 0.0%	0(0%) 0.0%	
	**Total,N = 145 (100%),Me = 32.59,SD = 7.106**		**64 (44.1%)**	**50(34.5%)**	**31(21.4%)**	
ANOVA				F(4,140) = 4.507,Sig.=0.002, Eta squared = 0.114		
Convex	Period of development	<= 5.00 (Group 1),N = 2(40%) Me = 25.00, SD = 1.414	2 (50%) 100.0%			0.361
		6.00–10.00( Group 2),N = 3 (60%) Me = 27.67, SD = 4.933	2(50%) 66.7%	1(100%) 33.3%		
	**Total,N = 5 (100%),Me = 26.60,SD = 3.847**		**4(80%)**	**1(20%)**		
ANOVA			F(1.3) = 0.505,Sig.=0.528			
Oblique	Period of deformity development	<= 5.00 (Group 1),N = 44 (26.7%) Me = 31.91,SD = 6.974	16(34.8%) 36.4%	22(28.9%) 50.0%	6(14.0%) 13.6%	0.240
		6.00–10.00(Group 2), N = 75(45.5%) Me = 35.41,SD = 7.398	22(47.8%) 29.3%	33(43.4% 44.0%	20(46.5%) 26.7%	
		11.00–15.00( Group 3),N = 37 (22.4%) Me = 35.57,SD = 7.526	7(15.2%) 18.9%	18(23.7%) 48.6%	12(27.9%) 32.4%	
		16.00–20.00(Group4),N = 6 (3.6%) Me = 37.50,SD = 7.232	1(2.2%) 16.7%	2(2.6%) 33.3%	3(7.0%) 50.0%	
		21.00 and more (Group 5),N = 3 (1.8%) Me = 38.67,SD = 4.163	0(0%) 0.0%	1(1.3%) 33.3%	2(4.7%) 66.7%	
	**Total,N = 165 (100%),Me = 34.65,SD = 7.400**		**46 (27.9%)**	**76 (46.1%)**	**43 (26.1%)**	
ANOVA			F(4,160) = 2.371,Sig.=0.055			

In the oblique shape of the MTC joint, 94.55% of patients appeared for treatment in the first 15 years after the presence of the deformity, in the transverse shape 82.76%, and in the convex shape all in the first ten years of development. Pearson’s Chi square independence test showed a statistically significant dependence of HVA and period of deformity development in the transverse form of MTC joint (Sig. = 0.013), while in other shapes the dependence was not statistically significant. (Table 5) The same test showed that the dependence of IMA and period development of deformity statistically significant only in the transverse form of the first MTC joint (Sig. = 0.049).

**Fig. 2:**
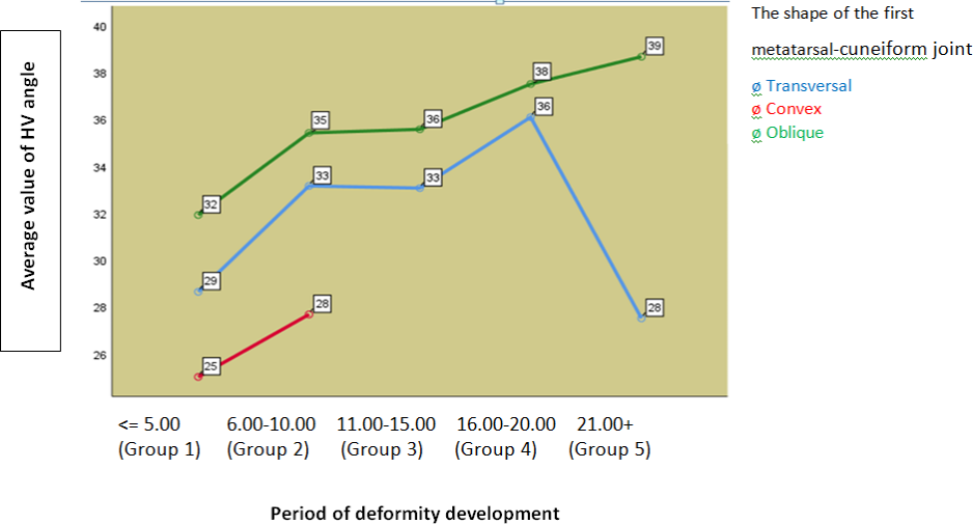
The ratio of mean HVA values to the shape of the I MTC joint and period of deformity development

The progression of HVA in this sample is most intense in the period from 5 to 10 years from the onset of symptoms, followed by a slower developmental course in the interval from 11 to 15 years, followed by a period of further deterioration, (Fig. 2) and (Table 5).

The analysis showed that the oblique shape of the MTC joint (six) was twice as common in persons younger than 18 years of age than in the transverse (three). In other age groups, the distribution is mostly even.

**Fig. 3:**
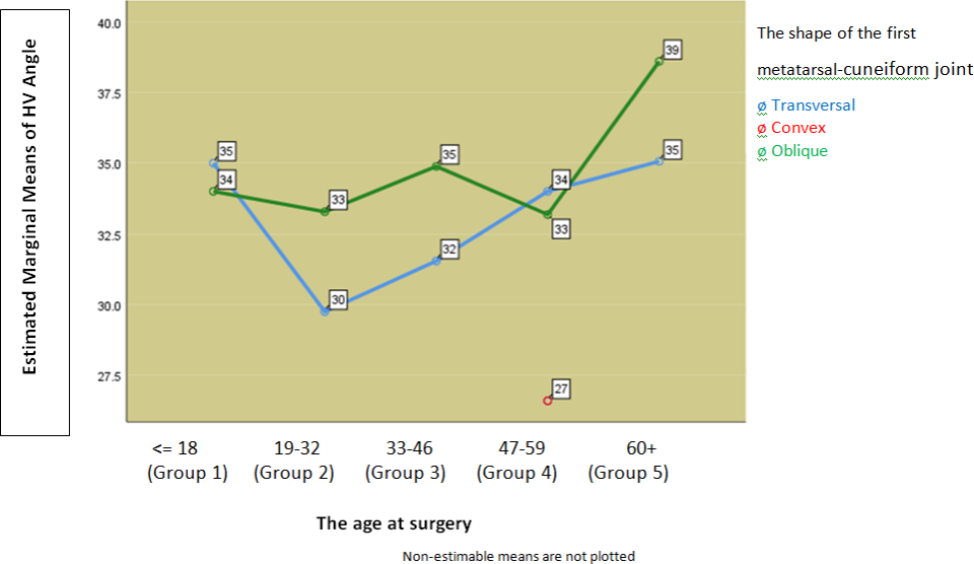
Average values of HVA according to the form of MTC joint in relation to the age of patients

Pearson’s Chi square test showed a statistically significant dependence of HVA on the shape of MTC joint in the age group from 33 to 46 years (p = 0.016), (Fig. 3).

## Discussion

Of the 315 feet treated in this study with severe HV deformity, the oblique shape of the first MTC joint was found in 165 or 52.4%, and the transverse shape in 145 feet or 46%. The oblique shape was dominated by moderate (46.1%) and severe (26.1%) degree of deformity measured by HVA in contrast to the transverse shape, which was dominated by mild deformity (44.1%). A statistically significant dependence of HVA and MTC joint shape was found (Sig. = 0.010). The influence of the shape of the joint on the value of HVA was investigated by one - factor analysis of variances. A statistically significant difference was found in the values of HVA in the three formed groups according to the form of the MTC joint, F (2,312) = 5,482, Sig. = 0.005 (Table 1). Subsequent comparison of Tukey’s HSD test of the actual difference between the mean values of the groups showed that the mean value of HVU in feet with an oblique shape (N = 165, Me = 34.65, SD = 7.4, 95% Cl: from 33.51 to 35.79, Min. = 18, Max. = 61) is statistically significantly different from the mean value of HVA in feet with the transverse shape (N = 145, Me = 32.59, SD = 7.106, 95% Cl: 31.43-33, 76, with Min. = 16, Max. = 50) with an average difference of R = -2.055, Sig. = 0.035, as well as the mean HVA in feet with a convex shape of the MTC joint (N = 5, Me = 26.6, SD = 3,847,95Cl: from 21.82 to 31.38, and Min. = 22 and Max = 31) with average difference R = 8.048, Sig. = 0.039. These results are consistent with previously published [12, 16] which found a significant dependence of HVA on the degree of stiffness and MTC of the joint.

The distribution of the subjects according to the severity of the deformity in relation to the IMA, in the oblique and the transverse shape is more even. However, the transverse shape is dominated by a mild degree (55.9%), and the oblique shape with a moderate degree of deformity (50.3%). Pearson’s Chi square test showed that the relationship between IMA values and MTC joint shape was not statistically significant (Sig. = 0.105). The influence of MTC joint shape on IMA values was investigated by a one-factor analysis of variance. Differences in mean IMA values in the three groups were not statistically significant (F (2,312) = 2,636, Sig. = 0.073) (Table 2). Published results of previous studies [9, 11] that dealt with similar issues, found a significant correlation between IMA and the angle of medial angulation of the first MTC joint, that was not confirmed by this study. In connection with the above said, we must emphasize the most important limitation of this study which refers to radiographic imaging procedure, with special emphasis on the position of the foot on which its radiographic appearance depends [18]. In addition to this, this study did not include analysis of the proximal articular surface of the I MT bone, the inclination of which directly affects the degree of I MT bone varisation and size of the IMA [19–22].

A statistically significant dependence of the position of the tibial sesamoid and HVA was found in both the transverse (Sig. = 0.002) and oblique shape of the MTC joint (Sig. = 0.000). One-factor analysis of variance of different groups showed the relationship between the position of the tibial sesamoid and HVA. A statistically significant difference was found between the average value of HVA formed groups according to the position of the tibial sesamoid both in the transverse (Sig. = 0.000, Eta squared = 0.188) and in the oblique shape (Sig. = 0.000 Eta squared = 0.172). (Table 3) Tukey’s HSD test of actual differences showed a statistically significant difference in the mean value of HVA in cases of ‘II’ position of the tibial sesamoid in relation to ‘III’ in the transverse and in the oblique shape of the I MTC joint. (Sig. = 0.000). These results show that the form of the I MTC of the joint does not affect the ratio of the position of the tibial sesamoid to the HVU value.

The relationship between the position of the tibial sesamoid and the value of IMA differs depending on the form of the I MTC joint. Pearson’s Chi square test found a statistically significant dependence of tibial sesamoid position and IMA (Sig. = 0.002) in the oblique shape, and one-factor analysis determined a statistically significant difference in IMA values between the formed groups according to sesamoid position (Sig. = 0.000, Eta squared = 0.107). The same test determined that in the transverse shape of the I MTC joint the described dependence was not statistically significant (Sig. = 0.102) while the one-factor analysis of variance showed the statistical significance of the difference of the IMA between the formed groups (Sig. = 0.030, Eta square = 0.048). 4) Tukey’s HSD test of actual differences showed a statistically significant difference in the mean value of IMA in cases of ‘II’ position of the tibial sesamoid in relation to ‘III’ in the oblique and transverse form of MTC joint (Sig. = 0.032). The above results of the analysis show that in the transverse form of the MTC joint, the change of the position of the tibial sesamoid is not accompanied by the expected increase of values of the IM angle. Dayton et al. [6] indicate that pronation of the I MT bone significantly contributes to the positioning of the sesamoid mechanism on the AP radiograph. Most researchers agree that the pronation of the I MT bone is the result of movement in the frontal plane at the level of the MTC joint [6, 23–25] but the possibility of torsion as a structural change of the I MT bone is also investigated [26]. This indicates the need to further examine the degree of pronation of the I MT bone in the transverse versus the oblique form and MTC joint.

HV is a progressive deformity and it develops faster in the oblique form of the MTC joint, because in the first 15 years after the presence of the deformity, 94.55% occurred due to treatment, and in the transverse 82.76%. In the transverse shape of the MTC joint, a statistically significant dependence of HVA and the period of deformity development was found (Sig. = 0.013, Table 5), as well as IMA (Sig. = 0.049), while in other shapes this dependence was not statistically significant. One-factor analysis of variance showed that in the transverse shape there is a statistically significant difference in HVA values between the formed groups of the period of deformity development (F (4,140) = 4,507, Sig. = 0,002), while in the oblique shape, during the early period of development, a moderate to severe deformity was achieved, so that this difference is not statistically significant (F (4,160) = 2.371, Sig. = 0.055) (Table 5).

Further analysis of the results showed that in persons under 18 years of age twice the prevalence of the oblique shape of the MTC joint (66.7%) compared to transverse (33.3%), and at the age of 60 and over where the oblique shape is also more prevalent (61.4%) compared to transversal (38.6%). In other age groups, the distribution is mostly even. Analyzing the dependence of HVA on the form of the first MTC joint in relation to the age of the subjects, we see that it is most pronounced in the age group from 33 to 46 years and that it is statistically significant (p = 0.016) (Fig. 3). One-factor analysis of variance showed that in the age group 33–46 years, the mean value of HVA in the oblique shape (N = 52, Me = 34.88, SD = 6.74.95% Cl: from 33.1 to 36.76) compared to transverse shape (N = 50, Me = 31.54, SD = 6.68.95% CL: from 29.64 to 33.44), statistically significantly different (Sig. = 0.013).

## Conclusion

The oblique shape of the first MTC joint is more common and leads to the development of a more severe form of HV deformity.

Values of HVA have a statistically significant correlation with the form of the first MTC joint. Statistically significant influence of the form of the first MTC joint on the size of the IMA was shown and its average values are significantly higher in the oblique form in relation to the transverse form of the joint.

Neither a statistically significant correlation nor statistically significant influence of the form of the first MTC joint on IMA was proven.

HVA values follow the position of the tibial sesamoid in both forms of the MTC joint while the size of the IMA in the transverse shape is not consistent with the change of position of this sesamoid.

HV deformity develops more rapidly in the feet with the oblique shape of the first MTC joint.

We believe that the oblique shape of the I MTC joint significantly contributes to the development of hallux valgus deformity.

## Data Availability

All data and materials of the research are in possession of the corresponding author.
